# Oral microbiota disorder in GC patients revealed by 2b-RAD-M

**DOI:** 10.1186/s12967-023-04599-1

**Published:** 2023-11-18

**Authors:** Shengfu He, Yating Sun, Weijie Sun, Mingyang Tang, Bao Meng, Yanyan Liu, Qinxiang Kong, Yongxiang Li, Jiawen Yu, Jiabin Li

**Affiliations:** 1 Department of Infectious Diseases, The First Affiliated Hospital of Anhui Medical University, Hefei, Anhui China; 2 Anhui Center for Surveillance of Bacterial Resistance, Hefei, Anhui China; 3 Institute of Bacterial Resistance, Anhui Medical University, Hefei, Anhui China; 4 Department of Infectious Diseases, Chaohu Hospital of Anhui MedicalUniversity, Hefei, Anhui China; 5 Department of Oncology, Anqing First People’s Hospital of Anhui Medical University/Anqing First People’s Hospital of Anhui Province, Anqing, China; 6 Department of General Surgery, The First Affiliated Hospital of Anhui Medical University, Hefei, China

**Keywords:** Gastric cancer, Oral fungi, Biomarker, 2b-RAD-M

## Abstract

**Background:**

Microbiota alterations are linked with gastric cancer (GC). However, the relationship between the oral microbiota (especially oral fungi) and GC is not known. In this study, we aimed to apply 2b-RAD sequencing for Microbiome (2b-RAD-M) to characterize the oral microbiota in patients with GC.

**Methods:**

We performed 2b-RAD-M analysis on the saliva and tongue coating of GC patients and healthy controls. We carried out diversity, relative abundance, and composition analyses of saliva and tongue coating bacteria and fungi in the two groups. In addition, indicator analysis, the Gini index, and the mean decrease accuracy were used to identify oral fungal indicators of GC.

**Results:**

In this study, fungal imbalance in the saliva and tongue coating was observed in the GC group. At the species level, enriched *Malassezia globosa* (*M*. *globosa*) and decreased *Saccharomyces cerevisiae* (*S*. *cerevisiae*) were observed in saliva and tongue coating samples of the GC group. Random forest analysis indicated that *M*. *globosa* in saliva and tongue coating samples could serve as biomarkers to diagnose GC. The Gini index and mean decreases in accuracy for *M*. *globosa* in saliva and tongue coating samples were the largest. In addition, *M*. *globosa* in saliva and tongue coating samples classified GC from the control with areas under the receiver operating curve (AUCs) of 0.976 and 0.846, respectively. Further ecological analysis revealed correlations between oral bacteria and fungi.

**Conclusion:**

For the first time, our data suggested that changes in oral fungi between GC patients and controls may help deepen our understanding of the complex spectrum of the different microbiotas involved in GC development. Although the cohort size was small, this study is the first to use 2b-RAD-M to reveal that oral *M*. *globosa* can be a fungal biomarker for detecting GC.

**Supplementary Information:**

The online version contains supplementary material available at 10.1186/s12967-023-04599-1.

## Introduction

The incidence of gastric cancer (GC) worldwide exceeds 1 million every year, resulting in nearly 800,000 deaths. Among all malignant tumors, it ranks fifth in incidence and has the third highest fatality rate [1]. *Helicobacter pylori* (*H. pylori*) was long thought to be the only microorganism that survives in the stomach and was classified as a Class I carcinogen by the World Health Organization (WHO) [2]. In fact, only 1–3% of people infected with *H. pylori* eventually develop GC [3]. With the development of sequencing technology, an increasing number of studies have confirmed the role of microorganisms other than *H. pylori* in the development of GC [4, 5]. The diagnosis of GC mainly relies on endoscopy. However, this is an invasive examination and is not widely available in some areas[6]. Therefore, researchers have been committed to finding noninvasive diagnostic methods for GC [7, 8]. Studies have confirmed that GC patients have a unique gastric microbiome compared to healthy people[9]. However, it is unnecessary to take gastric tissue for microbial examination to diagnose GC.

The oral microbiota is the second largest microecosystem after the gut microbiota, with more than 700 kinds of bacteria and over 70 kinds of fungi and viruses [10]. Every day, more than 1 L of saliva is secreted from the mouth and enters the stomach, which is accompanied by many microorganisms [11]. Since the acquisition of oral microorganisms is convenient and noninvasive, its great potential as a disease diagnostic marker has been gradually explored. Zhang et al. [12] confirmed that oral bacteria in colorectal cancer (CRC) patients were significantly different from those of the control group, and oral bacteria were good biomarkers for diagnosing CRC. Kawasaki et al. [13] found that oral microbes can be used as early diagnostic markers for esophageal cancer. Interestingly, large epidemiological studies have found that poor oral hygiene status is associated with an increased risk of GC [14]. Several studies have focused on the association between oral microorganisms and GC [15–17]. However, they all focused on oral bacteria and ignored oral microorganisms (such as fungi) other than bacteria. As an important member of the gut microbiota, fungi are involved in regulating immunoreactions and are associated with a variety of digestive tract tumors [18–20]. In 2021, Zhong et al. [21] performed ITS sequencing on GC tissue and adjacent normal tissue in 45 GC patients, and they found for the first time that the operational taxonomic unit (OTU) abundance of GC tissue was lower than that of adjacent normal tissue. After statistical analysis, it was found that gastric fungus can be used as a biomarker for the detection of GC. Their study opens up the possibility of using fungi as biomarkers for detecting GC.

The 2b-RAD sequencing for Microbiome (2b-RAD-M) method is a sequencing method for digesting genomic DNA of samples using type IIB restriction enzymes, producing equal-length DNA fragments after digestion, and amplifying, sequencing and positioning IIB digestion tags as species-specific 2b-RAD markers for microbial characterization and quantification. 16S ribosomal RNA (rRNA) and internal transcribed spacer (ITS) rDNA sequencing can only provide genus-level classification. On the other hand, 2b-RAD-M can allow for accurate identification to the species level. Metagenomic sequencing can be used to sequence the whole genome from a sample, but it is costly. Moreover, it requires a higher DNA concentration and is easily contaminated with host genetic information. However, 2b-RAD-M has been shown to be a low-cost technique that can be accurate to the species level and allows for the use of contaminated samples with low biomass and high host information [22–24].

This study is the first to use 2b-RAD-M to sequence saliva and tongue coating samples from GC patients and healthy people. Compared with the control group, there was a significant imbalance in the oral fungal community in GC patients. Oral* M*. *globosa* was found to be an indicator biomarker for detecting GC.

## Material and methods

### Participants

A total of 88 oral samples (44 saliva and 44 tongue coating) were collected from 44 patients who received an endoscopic examination at the First Affiliated Hospital of Anhui Medical University and were enrolled in this study. Twenty-six patients diagnosed with GC pathologically and eighteen healthy individuals were included. Twelve gastric tissues (6 tumor tissues and 6 adjacent normal tissues) were collected from 6 GC patients who underwent surgery at the First Affiliated Hospital of Anhui Medical University. The detailed information of 44 participants who provided oral specimens is shown in Table 1. The specific information of 6 patients who provided gastric tissue specimens is shown in Additional file 1: Table S1. The inclusion criteria were as follows: (1) adult male or female; (2) able and willing to provide saliva and tongue coating swab samples; and (3) able and willing to provide signed and dated informed consent. The exclusion criteria were as follows: (1) taking proton pump inhibitors (PPIs), antibiotics, probiotics, prebiotics, chemotherapy drugs, and any other drugs that can affect the oral microbiota within the last month; (2) malignant tumors in areas other than the stomach; (3) acute or chronic pulmonary, cardiovascular, hepatic, or renal disease; and (4) pregnancy or lactation. The study protocol was approved by the Committee on Medical Ethics of the First Affiliated Hospital of Anhui Medical University (approved no. PJ2023-07-77) adopting prospective specimen collection and retrospective blind evaluation (PRoBE) methods [25]. Approved guidelines were followed while performing the experiment. All patients were fully informed and signed informed consent forms.

**Table 1:** Demographic and clinical features of patients who provided oral specimens

Characteristics	GC(n = 26)	Control(n = 18)	*P* value
Age(years)	66.92 ± 5.66	65.00 ± 5.72	0.647
Sex			
Male	16 (61.5%)	11 (61.1%)	0.977
Female	10 (38.5%)	7 (38.9%)	
BMI (kg/m^2^)	21.21 ± 2.82	22.58 ± 2.09	0.087
Smoking status			
Never smoker	11 (42.3%)	9 (50.0%)	0.922
Former smoker	5 (19.2%)	3 (16.7%)	
Current smoker	10 (38.5%)	6 (33.3%)	
Alcohol consumption			
Never drink	15 (57.7%)	11 (61.1%)	0.830
< 1 standard drink/day	6 (23.1%)	5 (27.8%)	
≥ 1 standard drink/day	5 (19.2%)	2 (11.1%)	
Tumor site			
Upper third of stomach	10 (38.5%)	–	
Middle third of stomach	3 (11.5%)	–	
Lower third of stomach	13 (50.0%)	–	
Diameter (cm)	5.46 ± 2.21	–	
TNM stage			
I-II	6 (23.1%)	–	
III-IV	20 (76.9%)	–	
Differentiation			
Poor	15 (57.7%)	–	
Moderate	11 (42.3%)	–	

### Oral sample collection

All the participants were asked to fast overnight (≥ 8 h) and did not brush their teeth in the morning. Salivary and tongue coating sample collection and preparation were carried out in accordance with previously published consensus [26, 27]. Thirty minutes before sampling, participants were asked to rinse their mouths by gargling with sterile saline, and then 2 ml of saliva was collected in sterilized tubes. Each tongue coating sample was collected from the middle section of the tongue dorsum using a fresh one-off toothbrush and put into the test tube with saline. The tubes were centrifuged for 10 min at 3,000 r/min, and the precipitates were collected. All the samples were transported to the laboratory immediately with liquid nitrogen and then stored at − 80 °C.

### Tissue sample collection

Following surgical isolation of lesions, the samples comprising cancer tissue and adjacent normal tissues (with no abnormality on the mucosal surface, 5–10 cm from the tumor boundary) were transferred into sterile cryotubes, immediately transferred to the laboratory with liquid nitrogen, and then stored at − 80 °C until further use.

### DNA extraction, library preparation and 2b-RAD-M

A TIANamp Micro DNA Kit (Tiangen) was used to extract genomic DNA. The 2b-RAD-M library preparation method basically followed the original protocol according to previous studies [22, 28]. First, DNA was digested with 4 U of the enzyme BcgI (NEB) for 3 h at 37 °C. Subsequently, the adaptors were ligated to the DNA fragments. The ligation reaction system was as follows: 10 μl of digested product, 1 μl of 10 × T4 ligase buffer, each of the two adaptors at 0.2 µM, and 800 U of T4 DNA ligase (NEB). Ligation was carried out at 4 ℃ for 12 h. Then, the ligation products were amplified, and the PCR products were subjected to 8% polyacrylamide gel electrophoresis. A band of approximately 100 bp was cut from the polyacrylamide gel, and the DNA was diffused from the gel in nuclease-free water for 12 h at 4 °C. Sample-specific barcodes are introduced by PCR of primers with platform-specific barcodes. The PCR system per 20 μl was as follows: 7 μl ligation products, 4 μl 5 × HF buffer, 0.6 μl dNTP (10 mM), 0.4 μl of each primer, 0.2 μl Phusion high-fidelity DNA polymerase (2 U/µl), and 7.4 μl pure water. The PCR products were purified using the QIAquick PCR Purification Kit (Qiagen) and then sequenced using the Illumina Nova PE150 platform. All adaptor and primer sequences are provided in Additional file 1: Table S2. The DNA extraction of gastric tissue microbiota is shown in the Supplemental Materials.

## Data analysis

### Gastric tissue microbiota analysis

Detailed methods can be found in the Supplemental Materials.

### Sequencing processing and quantitative analysis of 2b-RAD-M

First, a total of 173,165 microbial genomes (including bacterial, fungal, and archaeal genomes) were obtained from the NCBI RefSeq database. Then, built-in Perl scripts were used to sample restriction fragments from microbial genomes by each of 16 type IIB restriction enzymes, which formed an enormous 2b-RAD microbial genome database. The set of 2b-RAD tags sampled from each genome was assigned under a GCF number, as well as GCF taxonomic information corresponding to the whole genome. Finally, all 2b-RAD tags from each GCF that occurred once within the genome were compared with those of all the others. Those 2b-RAD tags specific to a species-level taxon (having no overlap with other species) were developed as species-specific 2b-RAD markers, collectively forming a 2b-RAD marker database.

The G score value of each species was calculated using the following formula: G score _*species i*_ = $\sqrt {{\text{Si}} \times {\text{t}}i}$ (S: the number of reads assigned to all 2b-RAD markers belonging to species i within a sample; t: number of all 2b-RAD markers of species i that have been sequenced within a sample), and species with G scores above the threshold of 5 were screened as candidate species to control false positives. Next, the relative abundance of each species in the sample was calculated using the following formula: Relative abundance _*species i*_ =$\frac{Si/Ti}{{\sum\limits_{i = 1}^{n} {Si/Ti} }}$(S: the number of reads assigned to all 2b-RAD markers belonging to species i within a sample; T: number of all 2b-RAD markers of species i that have been sequenced within a sample) [22].

### Analysis of ecological characteristics of the salivary and tongue coating microbiota

Venn diagrams were used to present the unique and common bacterial species between the GC and control groups. The “vegan” package was used to calculate the alpha diversity index (Chao1, Shannon index, and Simpson index). Bray–Curtis distance, binary Jaccard distance, and Euclidean distance algorithms were computed to estimate the beta diversity using the “vegan” package. Linear discriminant analysis (LDA) effect size (LEfSe) was performed to identify taxa differentially represented between the GC side and the control side. We applied indicator analysis using the R software package to reveal the indicator species of each group and then performed statistical analysis of the indicator value between groups (*P* < 0.05 was selected by default). A random forest model was generated by the “randomForest” package to analyze key species that distinguish the GC group from the control group. To evaluate the discriminatory ability of the random forest model, a receiver operating characteristic curve (ROC) was constructed, and the area under the ROC curve (AUC) was calculated using the “pROC” package. Finally, the "igraph" and “ggraph” packages were used to plot the correlation between bacteria and fungi.

### Statistical analysis

R software (version 4.2.1) and SPSS (version 27) were used to perform all statistical analyses. Alpha diversity and microbial community comparisons were performed using the paired Wilcoxon test. The Bray‒Curtis distance, binary Jaccard distance, and Euclidean distance were statistically compared by permutational multivariate analysis of variance (PERMANOVA) to assess differences in beta diversity. The correlation at the fungal and bacterial phylum levels was calculated using Spearman correlation analysis based on the relative abundance. A *P* value < 0.05 was considered statistically significant.

## Results

### Comparison of bacteria and fungi in GC tissue and adjacent normal tissue

The study of Zhong et al. [21] was of great interest to us, so we also collected GC tissues and adjacent normal tissues from GC patients for 16S rRNA sequencing and ITS sequencing, respectively. As shown in Additional file 1: Fig S1A, GC tissue had a richer variety of bacteria than adjacent normal tissue. Interestingly, the number of fungal species in GC tissue was lower than that in adjacent normal tissue (Additional file 1: Fig S1B). Next, the abundance and distribution of bacteria and fungi in GC tissues and adjacent normal tissues were presented (Additional file 1: Fig S1C, S1D, S1E, and S1F). For bacteria, *Proteobacteria* was the most abundant phylum in GC tissue and adjacent normal tissue. Interestingly, the abundance of *Proteobacteria* in the GC group was lower. In contrast, *Firmicutes* and *Bacteroidetes* showed a higher abundance in GC tissues. Surprisingly, at the species level, we found that the abundance of *H. pylori* was higher in normal tissue than in GC tissue. Consistent with oral bacteria, compared with adjacent normal tissues, *Prevotella melaninogenica* (*P*. *melaninogenica*) was more abundant in GC tissues. For fungi, *Mucor racemosus* (*M*. *racemosus*) is the most abundant species in GC tissue, while *Wickerhamomyces anomalus* (*W. anomalus*) is the species with the highest abundance in adjacent normal tissue. To assess the alpha diversity of bacteria and fungi in GC tissues and adjacent normal tissues, we calculated the Chao1 index, Shannon index, and Simpson index separately. The Chao1 index of GC tissue bacteria was significantly higher than that in the adjacent normal tissue (*P* = 0.037), and although there was no significant difference between the two groups, the Shannon index and Simpson index were higher in GC tissue (Additional file 1: Fig S1G). The alpha diversity of fungi in GC tissues was lower than that in normal tissues adjacent to cancer, but the differences were not statistically significant (Additional file 1: Fig S1H).

### Saliva and tongue coating bacteria comparison between the GC and control groups

**Fig. 1:**
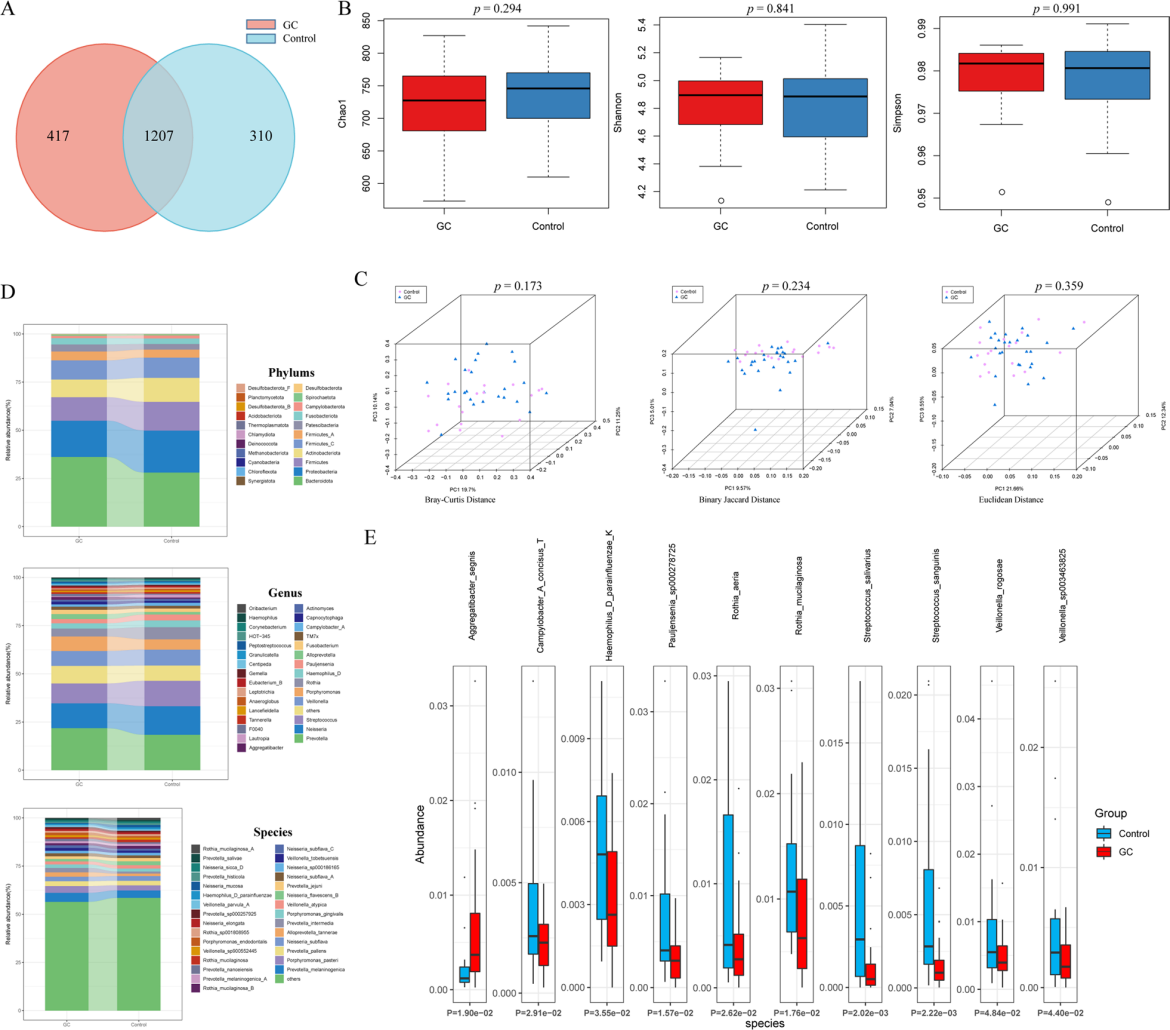
Comparison of salivary bacteria between gastric cancer patients and healthy controls. **A** Shared and unique species between the two groups shown by Venn diagram. **B** Comparison of alpha diversity (Chao1, Shannon index, and Simpson index) between the two groups. **C** Comparison of beta diversity (Bray–Curtis distance, Binary Jaccard distance and Euclidean distance) between the two groups. **D** The relative abundance and distribution of salivary bacteria at the phylum, genus, and species levels. **E** The top 10 species with different abundances between the two groups

As shown in the Venn diagram (Fig. 1A), there were 1207 (62.41%) identical species in the GC and control group salivary bacteria, 417 (21.56%) unique bacteria in the GC group saliva and 310 (16.03%) unique bacteria in the control group saliva. Tongue coating bacteria of the GC and control groups showed similar conditions (Additional file 1: Fig S2A). The Chao1, Shannon index and Simpson index of saliva bacteria showed no significant difference between the two groups (Fig. 1B). We evaluated the beta diversity of salivary bacteria based on Bray‒Curtis distance, binary Jaccard distance, and Euclidean distance, and there was no statistically significant difference (P > 0.05) (Fig. 1C). Interestingly, the Chao1 index of tongue coating bacteria in the control group was significantly lower than that in the GC group (*P* = 0.028), while there was no significant difference between the Simpson index and Shannon index (P > 0.05) (Additional file 1: Fig S2B). As shown in Additional file 1: Fig S2C, there were significant differences in beta diversity between the two groups based on Bray‒Curtis distance (*P* = 0.01), binary Jaccard distance (*P* = 0.001), and Euclidean distance (*P* = 0.026).

We further showed the distribution and relative abundance of bacteria in saliva and tongue coating samples from the GC and control groups at the phylum, genus, and species levels. As presented in Fig. 1D and Additional file 1: Fig S2D, the salivary and tongue coating bacteria in the GC and control groups were mainly dominated by *Bacteroidota*, *Proteobacteria*, *Actinobacteriota*, and *Firmicutes*. Compared to the control group, the relative abundance of *Bacteroidota* in both saliva and tongue coating of the GC group was significantly higher, while *Actinobacteriota* was significantly lower than that in the control group (Additional file 1: Fig S3). As shown in Fig. 1E, among the 10 bacterial species with the largest relative abundance differences, *Aggregatibacter segnis* (*A*. *segnis*) was the only species with a higher relative abundance in the saliva of the GC group (*P* < 0.05).

To explore whether salivary or tongue coating bacteria could be good biomarkers for GC, we performed indicator analysis and random forest analysis. As shown in Additional file 1: Fig S4 and Additional file 1: Fig S5, although the indicator analysis results showed that many bacterial species could be used as indicator species, *P. melaninogenica* had the largest mean decrease in accuracy for both salivary and tongue coating bacteria. Therefore, we constructed ROC curves for *P. melaninogenica* for saliva (Additional file 1: Fig S4C) and tongue coating (Additional file 1: Fig S5C). However, the results were unsatisfactory.

### Salivary fungal disorders in GC

**Fig. 2:**
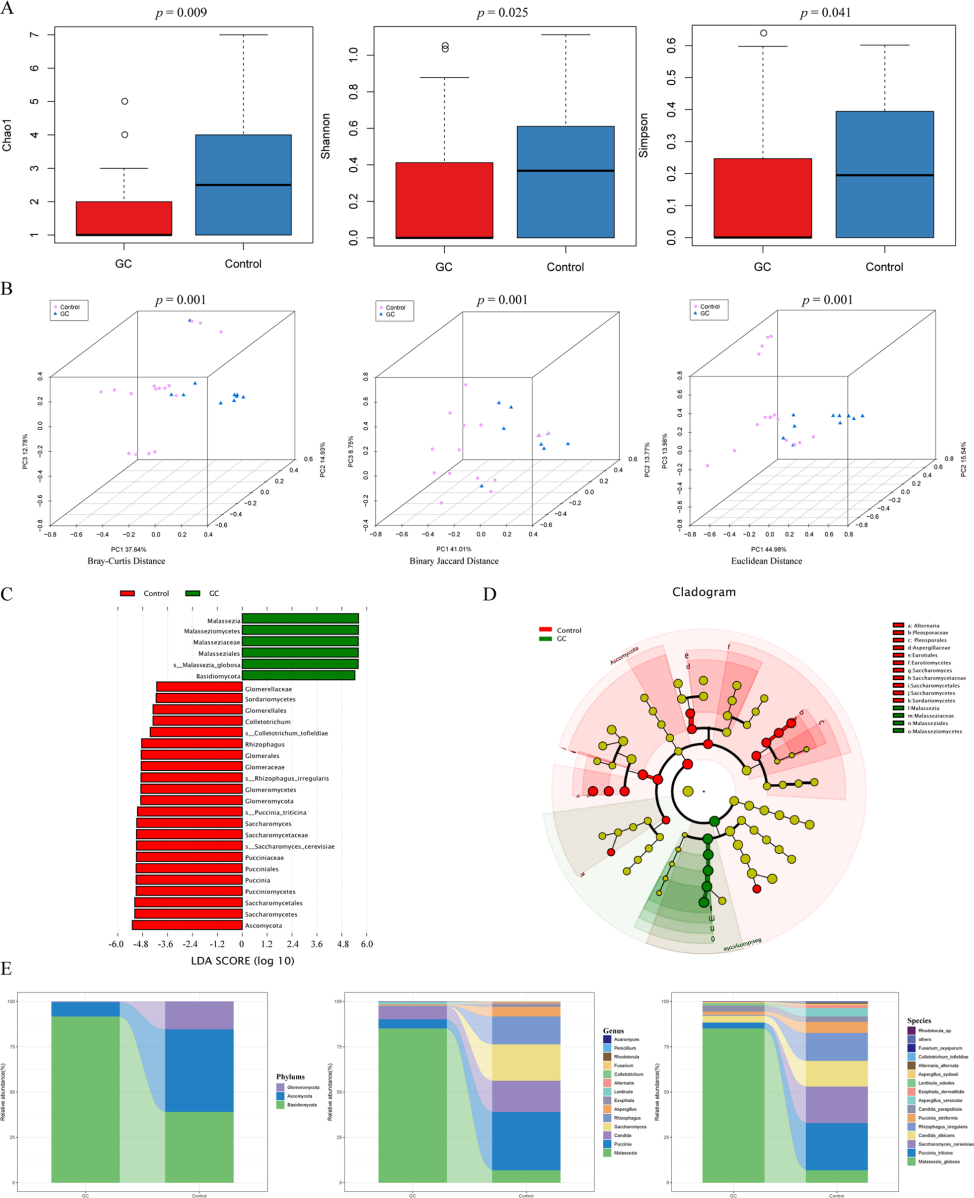
Salivary fungal diversity, abundance, and distribution in the two groups. **A** Comparison of salivary fungal alpha diversity between the two groups based on Chao1, Shannon index, and Simpson index. **B** Comparison of salivary fungal beta diversity (Bray–Curtis distance, binary Jaccard distance and Euclidean distance) between the two groups. **C, D** The LEfSe results identified the most divergent fungal taxa in the two groups and scored the two groups of saliva samples by LDA. The brightness of each point was proportional to the size of its effect. **E** The relative abundance of the salivary fungal phyla, the top 15 most abundant genera, and species is represented in the bar plot

The Chao1 index, Shannon index, and Simpson index were calculated to compare the alpha diversity of salivary fungi between the GC group and the control group. As shown in Fig. 2A, Chao1 (*P* = 0.009), Shannon (*P* = 0.025) and Simpson (*P* = 0.041) in the GC group were significantly lower than those in the control group. We evaluated the beta diversity of salivary fungi based on Bray‒Curtis distance, binary Jaccard distance, and Euclidean distance (Fig. 2B), with statistically significant differences between the two groups (*P* = 0.001). LEfSe was performed to identify different abundance taxa in the two groups. *M. globosa* was the fungal species with the highest abundance in the saliva of the GC group, and *Saccharomyces cerevisiae* (*S. cerevisiae*) was the most abundant fungal species in the saliva of the control group (Fig. 2C, D). Figure 2E shows the abundance and distribution of the two groups of salivary fungi at the phylum, genus, and species levels. *Basidiomycota* and *Ascomycota* were dominant in both the GC group and the control group, but the relative abundance of *Basidiomycota* in the GC group was significantly higher than that in the control group, and the abundance of *Ascomycota* was significantly lower than that in the control group (Additional file 1: Fig S6A). At the genus level, *Malassezia* abundance was significantly higher in the GC group than in the control group (Additional file 1: Fig S6B). The abundance of *M. globosa* in the saliva of the GC group was significantly higher than that in the control group. However, the abundance of *Saccharomyces cerevisiae* (*S. cerevisiae*) in the GC group was significantly lower than that in the control group (Additional file 1: Fig S6C).

**Fig. 3:**
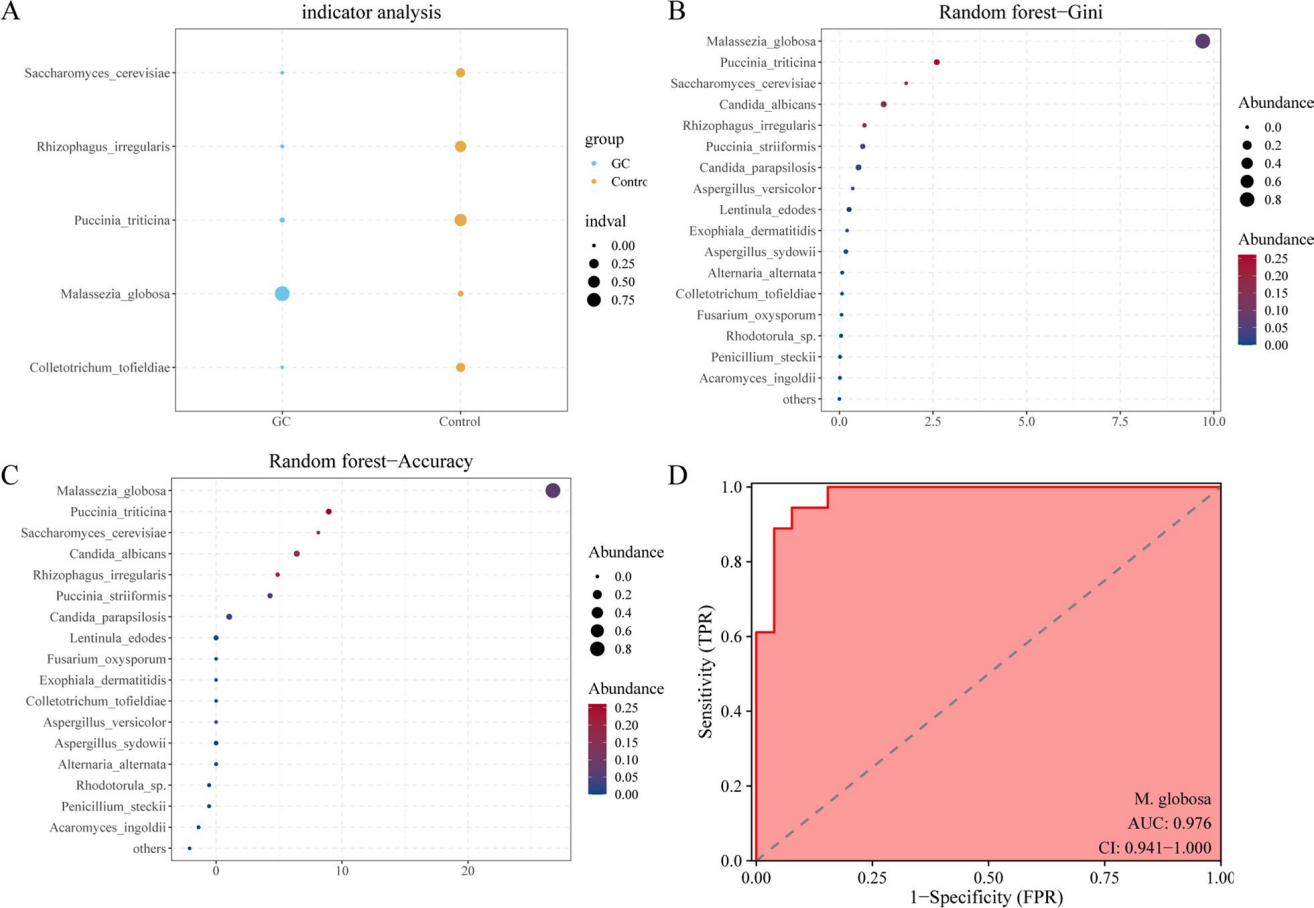
Salivary *M. globose* has a strong indication ability for GC. **A** The indicator analysis showed the ability of *M. globosa* to be an indicator of GC. **B, C** Both the mean decrease accuracy and the Gini index of salivary *M. globosa* were the largest. **D** Salivary *M. globosa* achieved an area under the receiver operating characteristic curve (AUC) of 0.976 for the classification of the GC group from the control group

Since *M. globosa* was significantly enriched in the saliva of the GC group, we hypothesized that salivary *M. globosa* can be used as a potential indicator biomarker for detecting GC. Random forest analysis showed that *M. globosa* had a potentially high GC indicator value (Fig. 3A). In addition, both the Gini index (Fig. 3B) and the mean decrease in accuracy (Fig. 3C) for *M. globosa* were the largest. ROC curve analysis was performed to assess the accuracy of saliva *M. globosa* in diagnosing GC, and an AUC value of 0.976 was observed. The above results all suggested that salivary *M. globosa* is an effective indicator for distinguishing GC patients and controls with a certain degree of accuracy.

### Tongue coating fungi comparison between GC and control

**Fig. 4:**
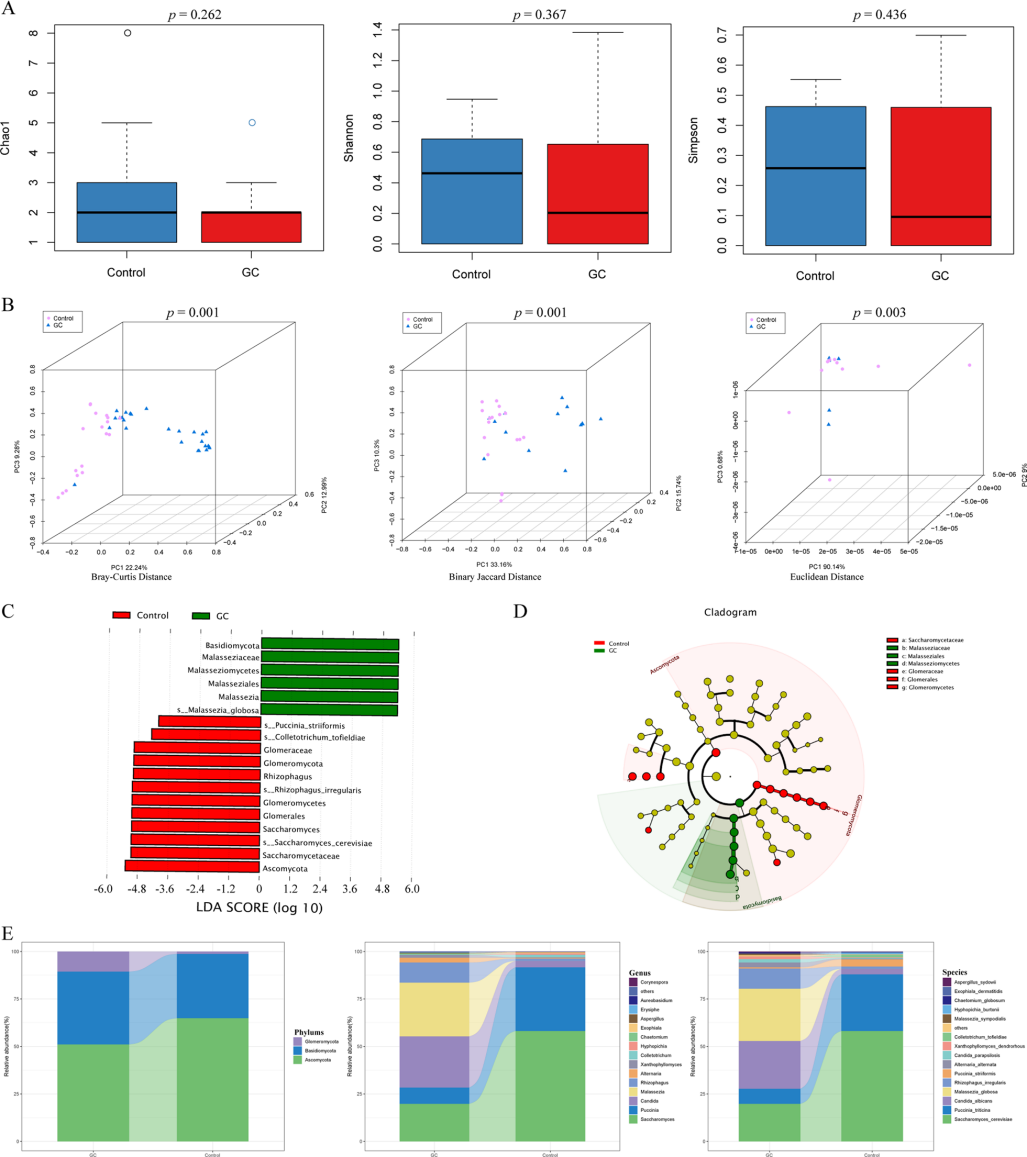
Tongue coating fungi diversity, abundance, and distribution in the two groups. **A** Comparison of tongue coating fungal alpha diversity between the two groups based on Chao1, Shannon index, and Simpson index. **B** Comparison of tongue coating fungal beta diversity (Bray–Curtis distance, binary Jaccard distance and Euclidean distance) between the two groups. **C**, **D** The LEfSe method identified the most divergent fungal taxa in the two groups and scored the two groups of tongue coating samples by LDA. The brightness of each point was proportional to the size of its effect. **E** The relative abundance of the tongue coating fungal phyla, the top 15 most abundant genera, and species is represented in the bar plot

We calculated the Chao1 index, Shannon index, and Simpson index to evaluate the alpha diversity of tongue coating fungi in the two groups. Although there was no statistically significant difference, the alpha diversity of tongue coating fungi in the GC group was lower than that in the control group (Fig. 4A). Meanwhile, the Bray‒Curtis distance (*P* = 0.001), binary Jaccard distance (*P* = 0.001), and Euclidean distance (*P* = 0.003) were again used to assess the beta diversity of the two groups (Fig. 4B). The LEfSe results were similar to those in saliva; *M. globosa* was the most abundant species of tongue coating fungi in the GC group, and the most abundant species in the control group was *S. cerevisiae* (Fig. 4C, D). We again showed the relative abundance and distribution of tongue coating fungi in the GC and control groups at the phylum, genus, and species levels (Fig. 4E). At the phylum level, the tongue coating fungi of both groups were dominated by *Basidiomycota* and *Ascomycota*. The relative abundance of *Ascomycota* in the GC group was significantly lower than that in the control group, while the abundance of *Basidiomycota* was significantly higher than that in the control group (Additional file 1: Fig S7A). To our surprise, we did not detect *M. globosa* in the tongue coating of the control group at the species level. Similar to saliva, the relative abundance of *S. cerevisiae* in the GC group was significantly lower than that in the control group (Additional file 1: Fig S7C).

**Fig. 5:**
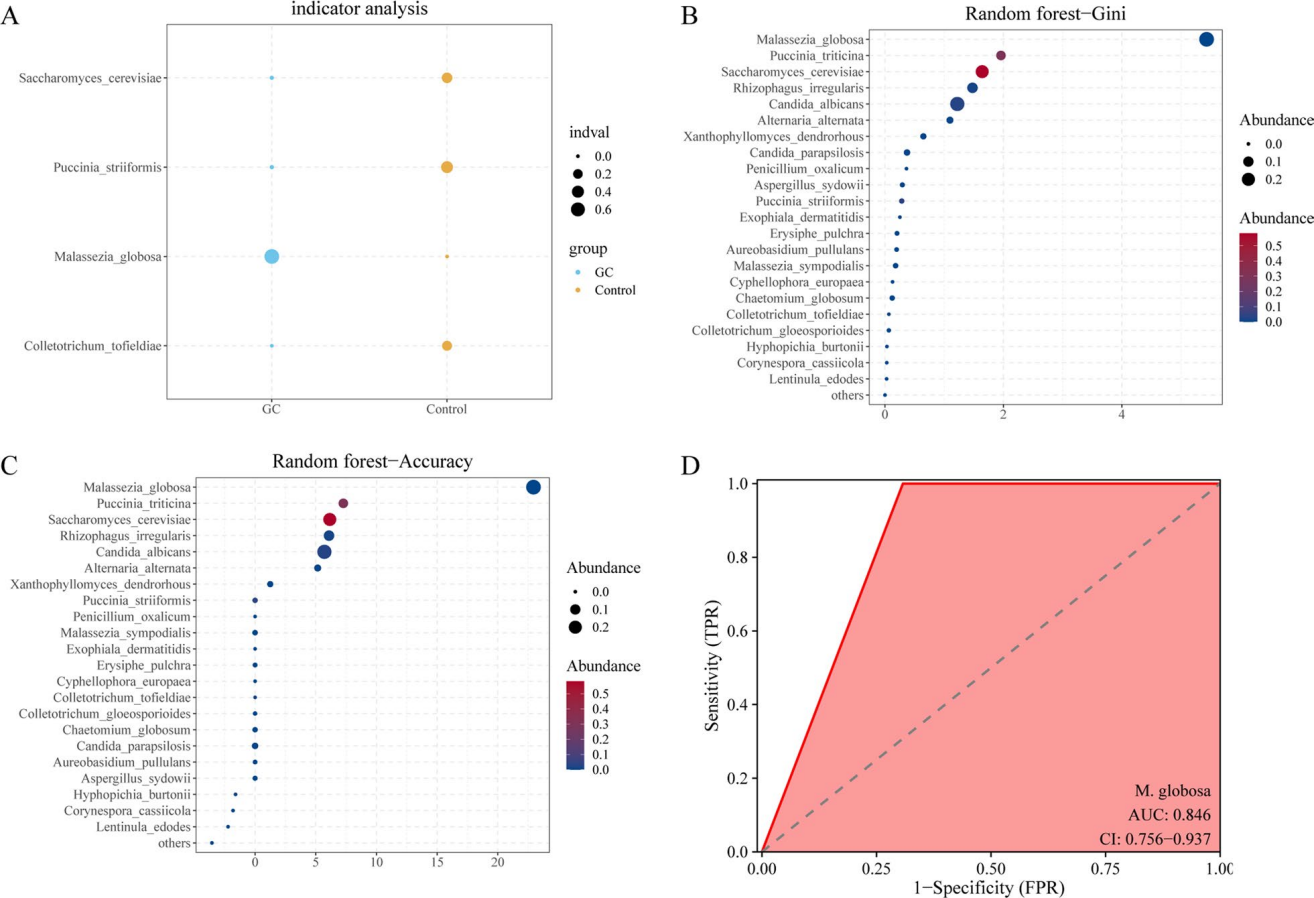
Tongue coating *M. globosa* has a strong indicator ability for GC. **A** The indicator analysis showed the ability of tongue coating *M. globosa* as an indicator of GC. **B**, **C** Both the mean decrease in accuracy and the Gini index of tongue coating *M. globosa* were the largest. **D** Tongue coating *M. globosa* achieved an area under the receiver operating characteristic curve (AUC) of 0.846 for the classification of the GC group from the control group

Similarly, we used random forest analysis for screening and found that tongue coating *M. globosa* was a potential GC indicator species (Fig. 5A), and both the Gini index (Fig. 5B) and mean decrease in accuracy (Fig. 5C) of tongue coating *M. globosa* were the largest. Subsequently, we applied the ROC curve to verify the ability of tongue coating *M. globosa* as a biomarker to diagnose GC and observed an AUC of 0.846 (Fig. 5D). All the results above suggested that tongue coating *M. globosa* has certain value as a biomarker for the diagnosis of GC.

### Ecological interactions among differentially abundant bacterial and fungal phyla

**Fig. 6:**
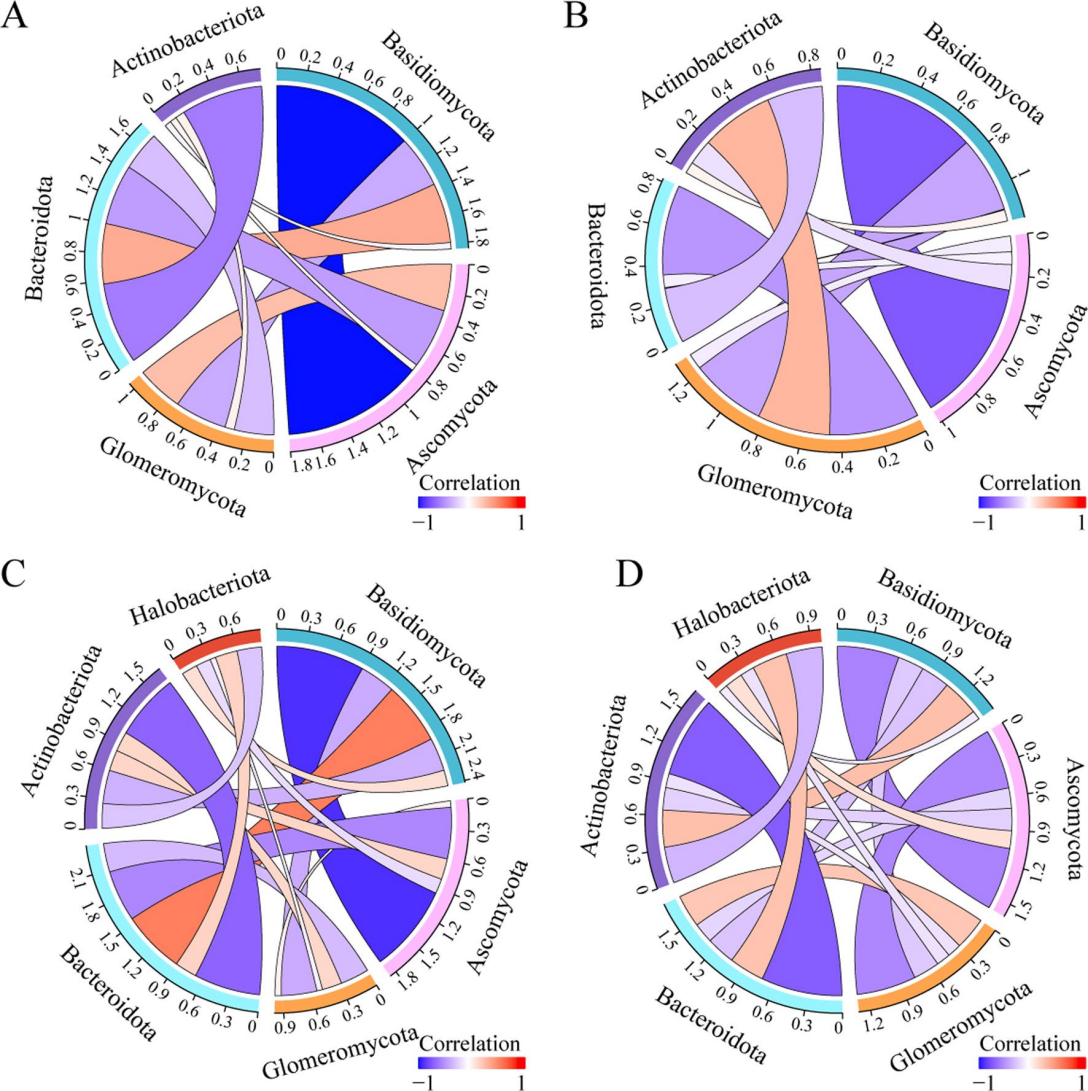
Perturbed intrakingdom and interkingdom ecological networks in gastric cancer (GC). The chord diagram of salivary fungi and bacteria with the greatest difference in relative abundance at the phylum level in the GC group (**A**) and the control group (**B**). The chord diagram of tongue coating fungi and bacteria with the greatest difference in relative abundance at the phylum level between the GC group (**C**) and the control group (**D**)

To demonstrate the interconnections between bacteria and fungi with significant differences between the GC and control groups, we constructed chordal diagrams of saliva and tongue coating bacteria versus fungi at the significantly different phylum level. Specifically, saliva and tongue coating *Basidiomycota* in the GC group were both positively correlated with *Bacteroidota* and negatively correlated with *Ascomycota* (Fig. 6A, C). Conversely, the phenomenon was reversed in the control group (Fig. 6B, D). This reflects the contribution of synergistic interkingdom interactions to oral microbiota homeostasis. Interestingly, the degree of negative correlation between *Basidiomycota* and *Ascomycota* was higher in both the saliva and tongue coating of the GC group than in the control group, which further demonstrated the imbalance of the oral fungal community in GC patients. In addition, saliva *Glomeromycota* was positively correlated with *Actinobacteriota* and negatively correlated with *Bacteroidota* in the control group (Fig. 6B). This phenomenon was significantly weakened in the GC group (Fig. 6A). Tongue coating *Halobacteriota* was negatively correlated with *Ascomycota* and positively correlated with *Basidiomycota* in the GC group, while the opposite phenomenon was observed in the control group (Fig. 6C, D). In summary, we showed the interaction between oral fungi and bacteria in the GC and control groups. An imbalance of interkingdom ecological co-occurrence relationships in saliva and tongue coating may be associated with GC.

## Discussion

Our study is the first to use 2b-RAD-M, a sequencing technology capable of characterizing microbiota at the species level with high accuracy and low cost, to illustrate the oral microbiota differences between GC patients and healthy people. We not only showed the changes in saliva and tongue coating bacteria but also showed for the first time the saliva and tongue coating fungal changes in GC patients compared with healthy controls. More importantly, we found that the relative abundance of *M. globosa* in the GC groups was significantly increased in both saliva and tongue coating. Oral *M. globosa* is an effective indicator species for detecting GC.

In this study, there were no statistically significant differences in the alpha diversity and beta diversity of salivary bacteria between the GC group and the control group. Notably, the salivary *A. segnis* of the GC group was significantly higher than that in the control group, which is consistent with the study of Jian Shu et al. [29]. They found that increased levels of *A. segnis* might be naturally regulated by salivary protein glycopatterns in GC patients. Strikingly, the Chao1 index of tongue coating bacteria in the GC group was significantly higher than that in the control group, and the beta diversity of the two groups significantly differed. This result indicated that the tongue coating bacteria in the GC group were significantly changed, which was consistent with previous studies [30]. The relative abundances of salivary and tongue *P. melaninogenica* in the GC group was higher than those in the control group. Surprisingly, we observed enrichment of *P. melaninogenica* in GC tissue compared to adjacent normal tissue, which was consistent with a previous study [31]. In addition, Zeng et al. [32] found that the proportion of *Prevotella* in oral cancer tissue was larger than that in normal tissue. Based on the above results, we hypothesize that oral *P. melaninogenica* may be a biomarker to distinguish GC from controls. Although random forest analysis suggested that the mean decrease in accuracy of both saliva and tongue coating *P. melaninogenica* was the largest of all bacteria, the ROC curve showed an AUC value of 0.620 for saliva *P. melaninogenica* and 0.536 for tongue coating *P. melaninogenica*. Whether orally enriched *P. melaninogenica* is associated with GC deserves further study.

Our results illustrated that the alpha diversity of salivary fungi in the GC group was significantly lower than that in the control group, and there was a significant difference in beta diversity. For tongue coating fungi, although there was no statistically significant difference in the alpha diversity correlation index between the two groups, a lower trend could be observed in the GC group. The beta diversity of the two groups significantly differed. These results also indicated that the abundance of fungi in the saliva and tongue coating of GC patients is lower than that of healthy controls within a single sample (alpha diversity). In addition, significant differences of fungal beta diversity in saliva and tongue coating between the two groups,

which also indicated significant changes in distribution of oral fungi in GC group compared with control group. At the phylum level, saliva and tongue coating fungi in both the GC group and the control group were mainly composed of *Ascomycota* and *Basidiomycota*, which was similar to the composition of gut fungi [33]. Intriguingly, the relative abundance of *Basidiomycota* in both the saliva and tongue coating of the GC group was significantly higher than that in the control group, whereas the relative abundance of *Ascomycota* in both the saliva and tongue coating was significantly lower than that in the control group. The increased ratio of *Basidiomycota* to *Ascomycota* reflects fungal dysbiosis [34]. These results suggested that the fungal community of the saliva and tongue coating in GC patients was disordered. To our surprise, *Malassezia* was significantly more abundant in the GC group than in the control group at the genus level, which is the most common fungal genus of human skin and can also colonize the gut [34]. In recent years, an increasing number of studies have found that gut *Malassezia* is associated with a variety of diseases. Gut *Malassezia* enables the release of proinflammatory cytokines (e.g., IL-6) through mast cell activation and regulation of the MAPK pathway, which exacerbates gut inflammation [35, 36]. Our results showed that the most abundant species of saliva- and tongue-coating fungi in the GC group was *M. globosa*. Previous studies have demonstrated that *M. globosa* can promote the development of pancreatic cancer by activating the MBL-C3 pathway [20]. In addition, oral pathogenic microbiota can exacerbate gastrointestinal inflammation directly or indirectly[37]. For example, oral pathogenic microbiota can colonize the gastrointestinal tract directly and exacerbate local inflammation [38, 39]. On the other hand, oral microbial-specific Th17 cells can migrate to the gastrointestinal tract in a targeted manner, and when oral microbial colonization is promoted, these Th17 cells can be specifically activated and aggravated by local inflammation [39]. Therefore, it is worth further exploring whether disordered oral fungi and enriched *M. globosa* are involved in the development of GC or are only manifestations after the occurrence of GC.

Another interesting result we found in this study was that *S. cerevisiae* was significantly reduced in the saliva and tongue coating of the GC group. *S. cerevisiae* is an important component of the human gut[33]. Related studies have confirmed that *S. cerevisiae* is a potential probiotic with the ability to improve gut barrier function, pathogen competitive rejection, antimicrobial peptide production, and immunomodulatory and nutritional effects [40, 41]. Our findings further highlight its potential as a probiotic. Whether *S. cerevisiae* can be used as a probiotic for GC prevention or treatment is likely to be the direction of our future research.

A growing number of studies have revealed the great potential of fungi as diagnostic markers for tumors [33, 42]. As mentioned above, the study of Zhong et al. [21] was of great interest to us. For the first time, they found that gastric tissue fungal imbalance is associated with GC and that gastric tissue *Candida albicans* (*C. albicans*) can be used as a biomarker for the diagnosis of GC. However, our results were different. *M*. *racemosus* is the fungus with the highest abundance in GC tissue, while *W. anomalus* is the most abundant fungus in adjacent normal tissues. Anthony Mannion et al. [43] performed metagenomic sequencing of 20 gastric tissues from high- and low-risk populations of GC in Colombia, South America, and found no significant differences in fungi. In addition, taking GC tissue for examination is an invasive procedure. Moreover, if you can obtain the patient’s gastric tissue, why not perform a more precise pathological examination? Saliva, as a “bridge” between the oral and gastrointestinal tract, has been found to serve as a biomarker library for a variety of diseases, including GC [17, 44]. Observing the change in tongue coating is an important part of traditional Chinese medicine diagnosis. Microbial changes in tongue coating have been observed in patients with GC [30, 45]. Unfortunately, these studies only focused on bacteria and ignored another important component, fungi. Our study demonstrated for the first time that saliva and tongue coating *M. globosa* may be potential diagnostic biomarkers for GC. The results undoubtedly provide a new direction for the noninvasive diagnosis of GC.

Normally, mucosal bacteria and fungi coexist and are in homeostasis [46]. The disorder of mucosal fungi could further lead to the imbalance of mucosal bacteria [47]. In turn, the maintenance of homeostasis of the mucosal fungal community is also dependent on the role of bacteria [48]. The disorder of mucosal bacteria in GC has been widely reported, and the disturbance of bacteria also promotes the progression of GC(4). Our study demonstrated the association between the bacterial and fungal phyla in the saliva and tongue coating of patients with GC and control groups, respectively. Our results showed that oral fungi and bacteria interacted with each other. Compared with the control group, homeostasis between fungi and bacteria in both saliva and tongue coating was broken in the GC group, and the correlation between some coexisting oral fungi and bacteria was weakened or even disappeared in the GC group. The results above suggested that oral bacteria at the stage of gastric cancer may be fungus dependent. Therefore, it is necessary to further study whether oral symbiotic bacteria-fungus homeostasis imbalance can promote the occurrence of gastric cancer. These results suggested that oral bacteria disturbances in GC may be fungus dependent. Therefore, it is necessary to further explore whether oral bacteria-fungus homeostasis imbalance is associated with GC in the future. In addition, we must acknowledge that our study has several further limitations. In particular, our study did not consider the effect of patients' dietary habits, especially a diet of highly processed or salty foods, which are considered predisposition factors for GC. In addition, several studies have shown that dietary habits can also affect oral microbiota. Therefore, in subsequent studies, we will further explore the influence of these factors on the oral microbiota (especially oral fungi).

## Conclusion

In conclusion, our study is the first to use 2b-RAD-M to sequence saliva and tongue coating microbiota from GC patients and healthy people. The bacterial and fungal communities of saliva and tongue coating in the two groups are shown. Disordered oral fungal communities in GC patients were first reported, and the great potential of saliva and tongue coating *M. globosa* as noninvasive biomarkers for detecting GC was discovered. Our results provide a possible new target for the diagnosis and treatment of GC.

### Supplementary Information

**Additional file 1:** **Figure S1.** Diversity, abundance, and distribution comparison of bacteria and fungi between gastric cancer (GC) tissues and adjacent normal tissues. Venn diagram showed the shared and unique bacteria(A) and fungi(B) species between GC tissues and adjacent normal tissues. Bacterial(C) and fungal(D) abundance and distribution in the two groups showed by barplot at phylum, genus, and species level. The composition of bacteria(E) and fungi(F) of GC tissues and adjacent normal tissues is shown by heat maps. The bacteria(G) and fungi(H) alpha diversity (Chao1, Shannon index, and Simpson index) of GC tissues and adjacent normal tissues. **Figure S2**. Comparison of tongue coating bacteria between gastric cancer(GC) patients and healthy controls. A, Shared and unique species between the two groups presented by Venn diagram. B, Comparison of alpha diversity (Chao1, Shannon index, and Simpson index) between the two groups. C, Comparison of beta diversity (Bray–Curtis distance, Binary Jaccard distance and Euclidean distance) between the two groups. D, The relative abundance and distribution of salivary bacteria at phylum, genus, and species level. E, The top 10 species with different abundance between the two groups. **Figure S3**. Comparison of the relative abundance of salivary(A) and tongue coating(B) bacteria at the phylum level between the two groups. **Figure S4.** Analysis of salivary bacteria as biomarkers for the diagnosis of gastric cancer (GC). A, Indicator analysis of salivary bacteria between the two groups. B, The mean decrease accuracy of salivary *Prevotella melaninogenica *was the largest. C, The salivary *Prevotella melaninogenica* achieved an area under the receiver operating characteristic curve (AUC) of 0.620 for the classification of the GC group from the control group. **Figure S5.** Analysis of tongue coating bacteria as biomarkers for the diagnosis of gastric cancer (GC). A, Indicator analysis of tongue coating bacteria between the two groups. B, The mean decrease accuracy of tongue coating *Prevotella melaninogenica *was the largest. C, The tongue coating *Prevotella melaninogenica* achieved an area under the receiver operating characteristic curve (AUC) of 0.536 for the classification of the GC group from the control group. **Figure S6.** Comparison of relative abundance of salivary fungi by Analysis of Variance (ANOVA) at phylum(A), genus(B), and species(C) level. **Figure S7.** Comparison of relative abundance of tongue coating fungi by Analysis of Variance (ANOVA) at phylum(A), genus(B), and species(C) level. **Table S1. **Demographic and clinical features of patients who provided gastric specimens. **Table S2. **Adaptors and primers used for 2bRAD-M library preparation.

## Data Availability

The datasets used and/or analyzed during the current study are available from the corresponding author on reasonable request.

## Abbreviations

GC: Gastric cancer
2b-RAD-M: 2B-RAD sequencing for Microbiome
*M*. *globosa*: *Malassezia globosa*
*S*. *cerevisiae*: *Saccharomyces cerevisiae*
AUC: Area under the receiver operating curve
*H. pylori*: *Helicobacter pylori*
WHO: The World Health Organization
CRC: Colorectal cancer
ITS: Internal transcribed spacer
OTU: Operational taxonomic units
16S rRNA: 16S ribosomal RNA
*P*. *melaninogenica*: *Prevotella melaninogenica*
*M*. *racemosus*: *Mucor racemosus*
*W. anomalus*: *Wickerhamomyces anomalus*
ROC: Receiver operating characteristic curve
LDA: Linear discriminant analysis
