# Supplementary material for: Oral microbiota disorder in GC patients revealed by 2b-RAD-M
Source: J Transl Med. 2023 Nov 18;21:831. doi: 10.1186/s12967-023-04599-1 (PMC10656981; doi:10.1186/s12967-023-04599-1)
Supplement: Supplementary file 1 — Additional file 1: Figure S1. Diversity, abundance, and distribution comparison of bacteria and fungi between gastric cancer (GC) tissues and adjacent normal tissues. Venn diagram showed the shared and unique bacteria(A) and fungi(B) species between GC tissues and adjacent normal tissues. Bacterial(C) and fungal(D) abundance and distribution in the two groups showed by barplot at phylum, genus, and species level. The composition of bacteria(E) and fungi(F) of GC tissues and adjacent normal tissues is shown by heat maps. The bacteria(G) and fungi(H) alpha diversity (Chao1, Shannon index, and Simpson index) of GC tissues and adjacent normal tissues. Figure S2. Comparison of tongue coating bacteria between gastric cancer(GC) patients and healthy controls. A, Shared and unique species between the two groups presented by Venn diagram. B, Comparison of alpha diversity (Chao1, Shannon index, and Simpson index) between the two groups. C, Comparison of beta diversity (Bray–Curtis distance, Binary Jaccard distance and Euclidean distance) between the two groups. D, The relative abundance and distribution of salivary bacteria at phylum, genus, and species level. E, The top 10 species with different abundance between the two groups. Figure S3. Comparison of the relative abundance of salivary(A) and tongue coating(B) bacteria at the phylum level between the two groups. Figure S4. Analysis of salivary bacteria as biomarkers for the diagnosis of gastric cancer (GC). A, Indicator analysis of salivary bacteria between the two groups. B, The mean decrease accuracy of salivary Prevotella melaninogenica was the largest. C, The salivary Prevotella melaninogenica achieved an area under the receiver operating characteristic curve (AUC) of 0.620 for the classification of the GC group from the control group. Figure S5. Analysis of tongue coating bacteria as biomarkers for the diagnosis of gastric cancer (GC). A, Indicator analysis of tongue coating bacteria between the two groups. B, The mea [file 12967_2023_4599_MOESM1_ESM.docx]

**Additional file Materials**


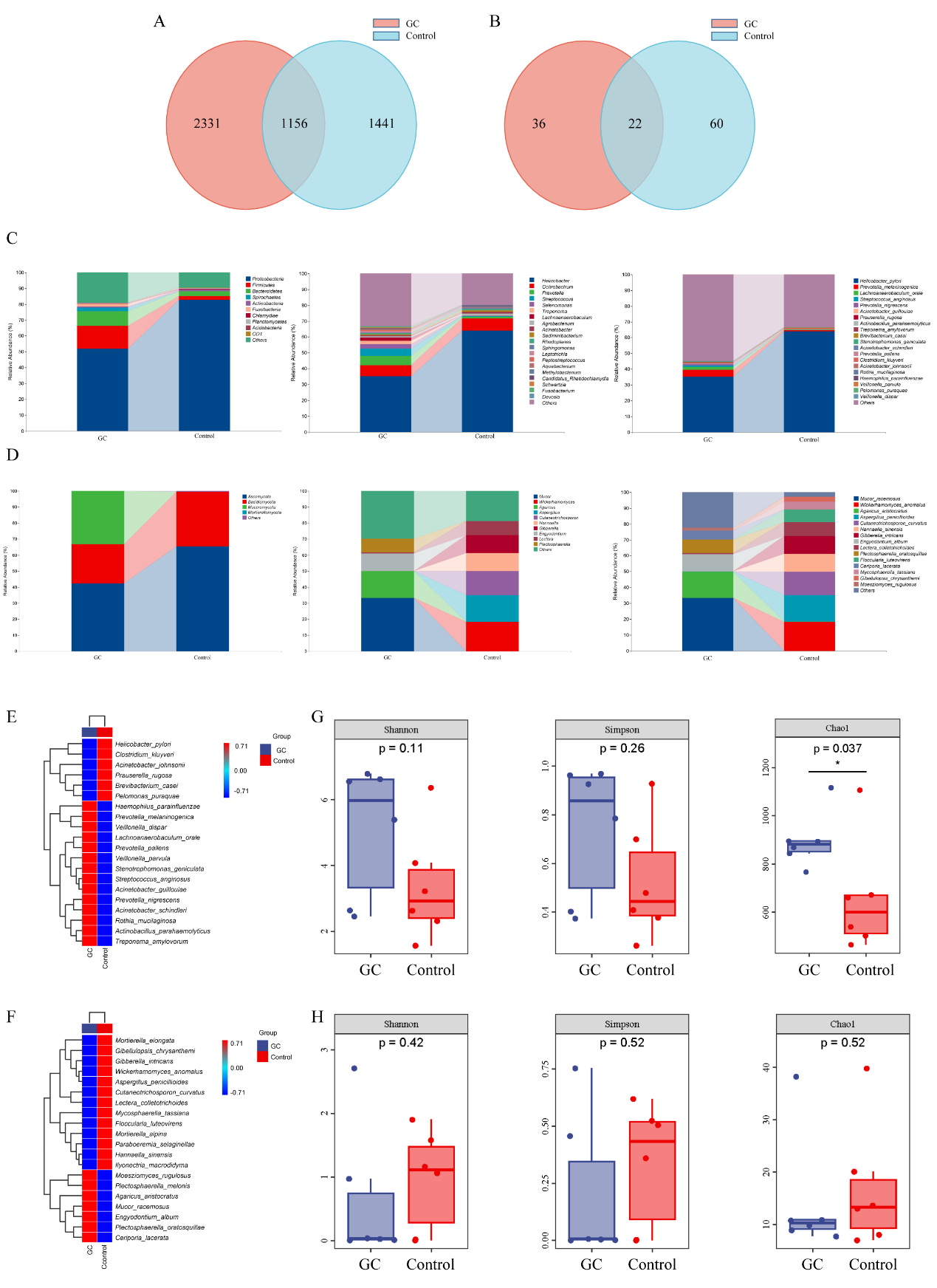


**Additional file 1: Figure S1.** Diversity, abundance, and distribution comparison of bacteria and fungi between gastric cancer (GC) tissues and adjacent normal tissues. Venn diagram showed the shared and unique bacteria(A) and fungi(B) species between GC tissues and adjacent normal tissues. Bacterial(C) and fungal(D) abundance and distribution in the two groups showed by barplot at phylum, genus, and species level. The composition of bacteria(E) and fungi(F) of GC tissues and adjacent normal tissues is shown by heat maps. The bacteria(G) and fungi(H) alpha diversity( Chao1, Shannon index, and Simpson index) of GC tissues and adjacent normal tissues.


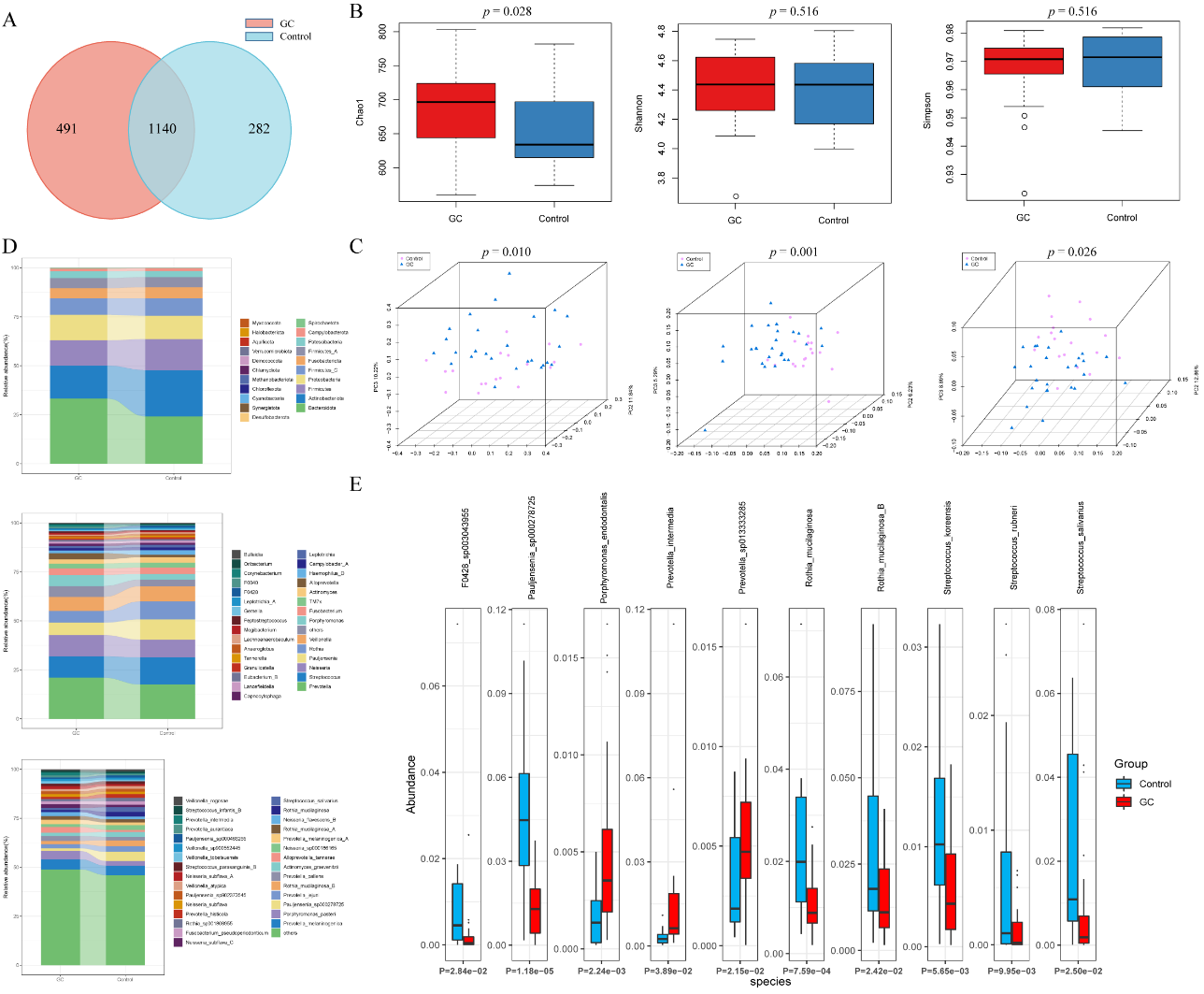


**Additional file 1: Figure S2**. Comparison of tongue coating bacteria between gastric cancer(GC) patients and healthy controls. A, Shared and unique species between the two groups presented by Venn diagram. B, Comparison of alpha diversity (Chao1, Shannon index, and Simpson index) between the two groups. C, Comparison of beta diversity (Bray–Curtis distance, Binary Jaccard distance and Euclidean distance) between the two groups. D, The relative abundance and distribution of salivary bacteria at phylum, genus, and species level. E, The top 10 species with different abundance between the two groups.


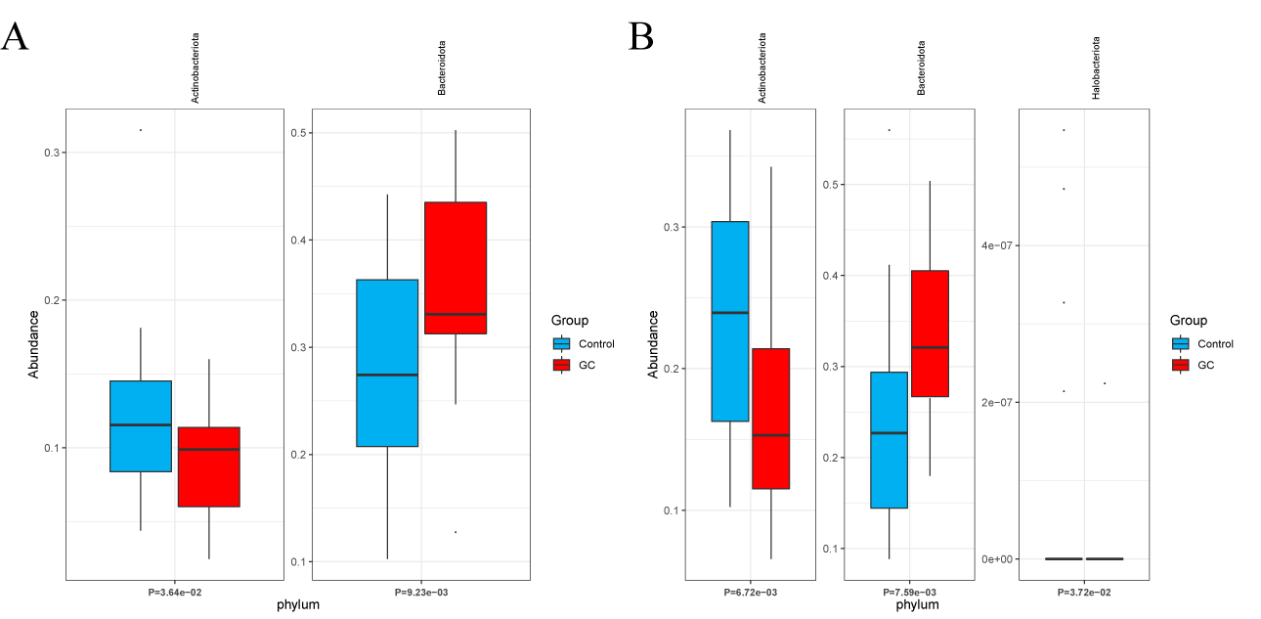


**Additional file 1: Figure S3**. Comparison of the relative abundance of salivary(A) and tongue coating(B) bacteria at the phylum level between the two groups.


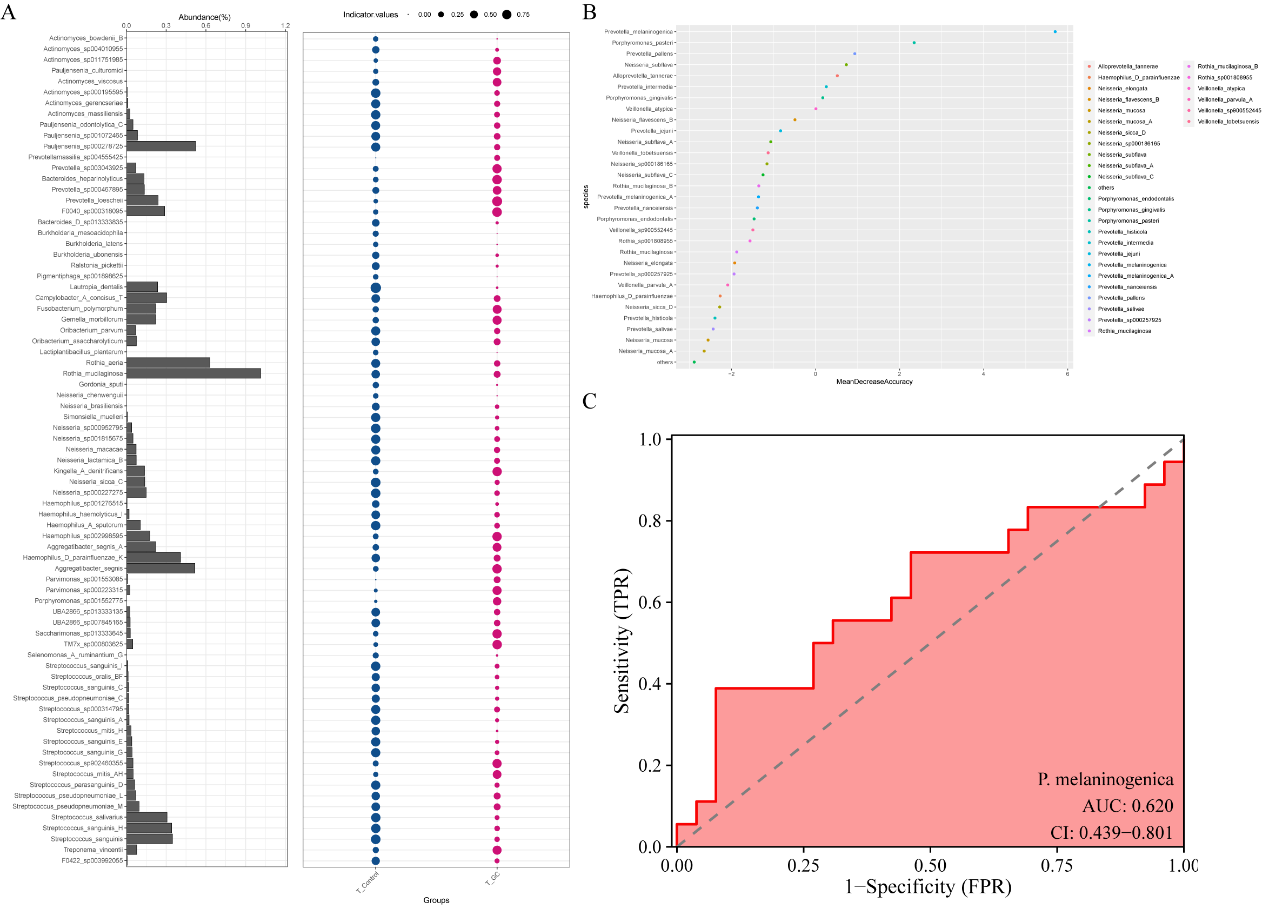


**Additional file 1: Figure S4.** Analysis of salivary bacteria as biomarkers for the diagnosis of gastric cancer (GC). A, Indicator analysis of salivary bacteria between the two groups. B, The mean decrease accuracy of salivary *Prevotella melaninogenica* was the largest. C, The salivary *Prevotella melaninogenica* achieved an area under the receiver operating characteristic curve (AUC) of 0.620 for the classification of the GC group from the control group.


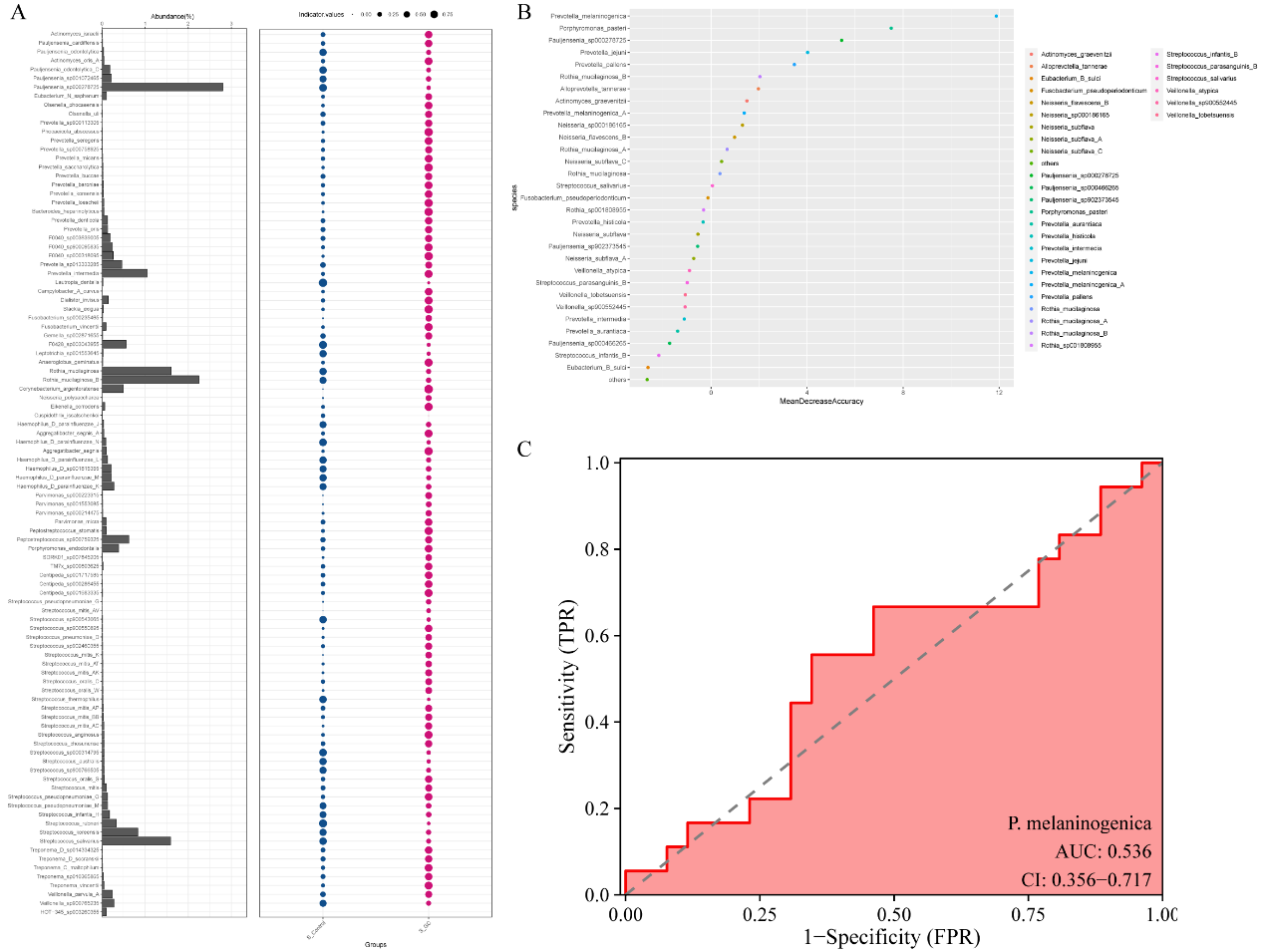


**Additional file 1: Figure S5.** Analysis of tongue coating bacteria as biomarkers for the diagnosis of gastric cancer (GC). A, Indicator analysis of tongue coating bacteria between the two groups. B, The mean decrease accuracy of tongue coating *Prevotella melaninogenica* was the largest. C, The tongue coating *Prevotella melaninogenica* achieved an area under the receiver operating characteristic curve (AUC) of 0.536 for the classification of the GC group from the control group.


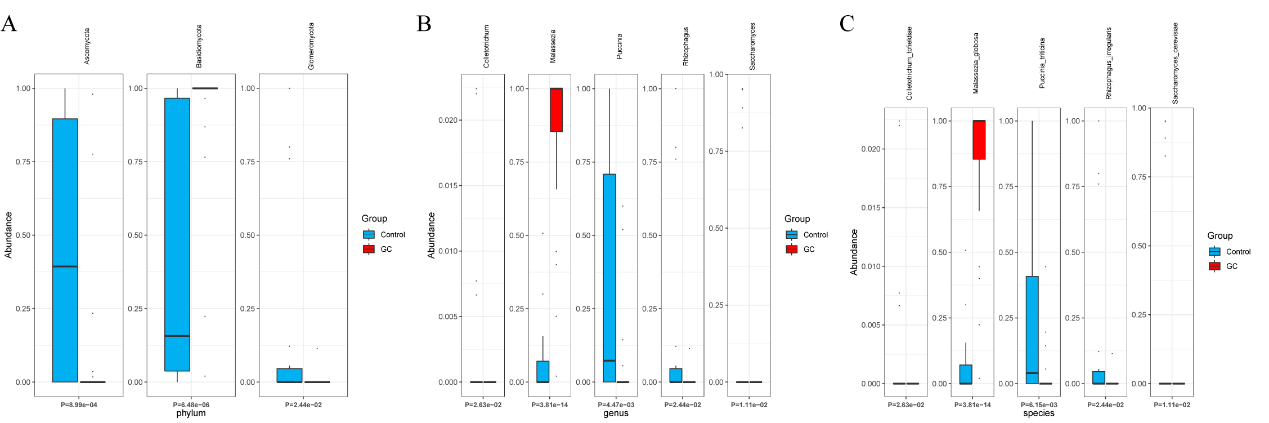


**Additional file 1: Figure S6.** Comparison of relative abundance of salivary fungi by Analysis of Variance (ANOVA) at phylum(A), genus(B), and species(C) level.


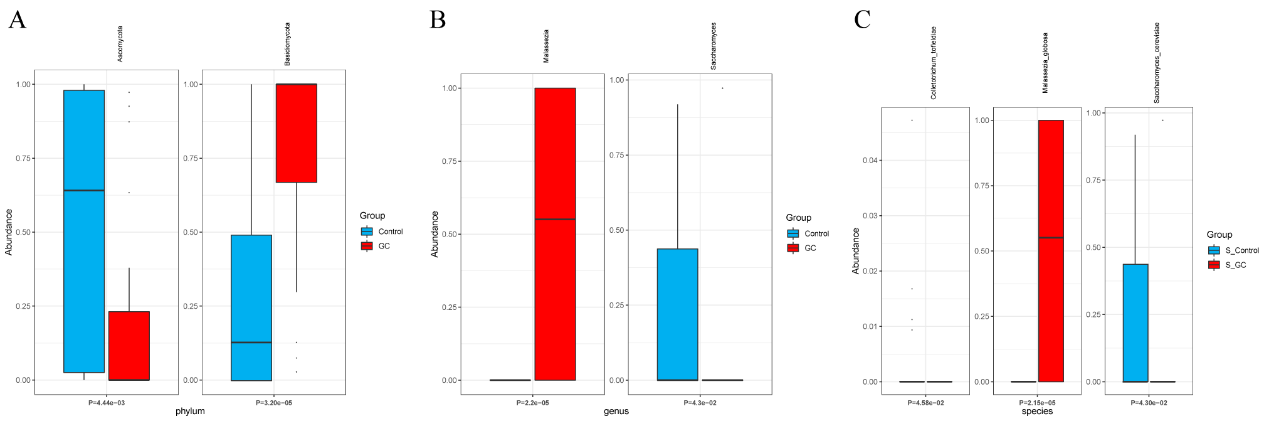


**Additional file 1: Figure S7.** Comparison of relative abundance of tongue coating fungi by Analysis of Variance (ANOVA) at phylum(A), genus(B), and species(C) level.

**Additional file 1 Table S1.** Demographic and clinical features of patients who provided gastric specimens

| Sample | | GC-01 | GC-02 | GC-03 | GC-04 | GC-05 | GC-06 |
| --- | --- | --- | --- | --- | --- | --- | --- |
| Age (year) | | 66 | 79 | 66 | 73 | 59 | 56 |
| Sex | | F | M | M | F | M | F |
| BMI (kg/m^2^) | | 28.07 | 22.32 | 20.83 | 26.06 | 26.64 | 24.46 |
| Tumor site  1.upper third of stomach  2.middle third of stomach  3.lower third of stomach | | 1 | 3 | 1 | 2 | 2 | 3 |
| Diameter (cm) | 8.0 | | 3.5 | 5.0 | 5.0 | 3.5 | 4.0 |
| Differentiation | | Moderate | Poor | Poor | Poor | Poor | Moderate |
| Nerve infiltration | | Yes | Yes | No | Yes | No | Yes |
| Lymph node metastasis | | Yes | Yes | No | Yes | No | Yes |
| Distant metastasis | | No | No | No | No | No | No |
| TNM (AJCC 8th) | | III | III | IIB | III | IIB | III |

**Additional file 1: Table S2.** Adaptors and primers used for 2bRAD-M library preparation.

| Adaptors and primers | Sequence (5’ to 3’) |
| --- | --- |
| Adap-1 sense | ACACTCTTTCCCTACACGACGCTCTTCCGATCTNNN |
| Adap-1 antisense | AGATCGGAAGAGC(AminoC6) |
| Adap-2 sense | GTGACTGGAGTTCAGACGTGTGCTCTTCCGATCTNNN |
| Adap-2 antisense | AGATCGGAAGAGC(AminoC6) |
| *Primers* |  |
| Primer1 | ACACTCTTTCCCTACACGACGCT |
| Primer2 | GTGACTGGAGTTCAGACGTGTGCT |
| Primer3 | AATGATACGGCGACCACCGAGATCTACACTCTTTCCCTACACGACGCT |
| Index primer | CAAGCAGAAGACGGCATACGAGATXXXXXXGTGACTGGAGTTCAGACGTGT |

**Gastric tissue microbiota analysis**

**DNA Extraction**

Total genomic DNA samples were extracted using the OMEGA Soil DNA Kit (M5635-02) (Omega Bio-Tek, Norcross, GA, USA), following the manufacturer’s instructions, and stored at -20 °C prior to further analysis. The quantity and quality of extracted DNAs were measured using a NanoDrop NC2000 spectrophotometer (Thermo Fisher Scientific, Waltham, MA, USA) and agarose gel electrophoresis, respectively.

**16S rRNA and ITS Gene Amplicon Sequencing**

The V3-V4 hypervariable regions of the 16S rRNA gene were amplified by polymerase chain reaction (PCR) using the following primers: 338F (5’-ACTCCTACGGGAGGCAGCA-3’) AND 806R (5’-GGACTACHVGGGTWTCTAAT-3’). The internal transcribed spacer (ITS) of the ITS1 region were amplified by PCR using the following primers:F(5’-GGAAGTAAAAGTCGTAACAAGG-3’) AND R(5’-GCTGCGTTCTTCATCGATGC-3’). Sample specific 7-bp barcodes were incorporated into the primers for multiplex sequencing. The reaction volume (25μl) is comprised of 5x reaction buffer (5μl), 5U/μl Fast pfu DNA Polymerase (0.25μl), 2.5mM dNTPs (2μl), 10μM each primer (1μl), DNA Template (1μl), and ddH2O (14.75μl). Cycling process consisted of initial denaturation at 98℃ for 5 min, followed by 25 cycles of denaturation at 98℃ for 30 s, annealing at 53℃ for 30 s, extension at 72℃ for 45 s, with a final extension at 72℃ for 5 min. PCR amplicons were purified with Vazyme VAHTSTM DNA Clean Beads (Vazyme, China), and quantified using the Quant-iT PicoGreen dsDNA Assay Kit (Invitrogen, USA). Quantified amplicons were pooled in equal amounts and pair-end 2x250 bp sequencing was performed using the Illumina NovaSeq platform with NovaSeq 6000 SP Reagent Kit (500 cycles) (Shanghai Personal Biotechnology Co., Ltd, China).

**Bioinformatic analysis**

Microbiome bioinformatic analysis was performed by QIIME2 2019.4 with slight modification according to the official tutorials[1]. Briefly, raw sequence data were demultiplexed using the demux plugin following by primers cutting with cutadapt plugin[2]. Sequences were then quality filtered, denoised, merged and chimera removed using DADA2[3]. Non-singleton amplicon sequences variants (ASVs) were aligned with mafft and used to construct a phylogeny with fasttree2[4, 5]. Taxonomy was assigned to ASVs using the classify-sklearn naïve Bayes taxonomy classifier in feature-classifier against the Greengenes 13.8[6, 7].

**Referrence**

1 Bolyen E.; Rideout J.R.; Dillon M.R.; et al. Reproducible, interactive, scalable and extensible microbiome data science using QIIME 2. Nat Biotechnol 2019;37:852-7.

2 Kechin A.; Boyarskikh U.; Kel A.; et al. cutPrimers: A New Tool for Accurate Cutting of Primers from Reads of Targeted Next Generation Sequencing. J Comput Biol 2017;24:1138-43.

3 Callahan B.J.; McMurdie P.J.; Rosen M.J.; et al. DADA2: High-resolution sample inference from Illumina amplicon data. Nat Methods 2016;13:581-3.

4 Katoh K.; Misawa K.; Kuma K.; et al. MAFFT: a novel method for rapid multiple sequence alignment based on fast Fourier transform. Nucleic Acids Res 2002;30:3059-66.

5 Price M.N.; Dehal P.S.; Arkin A.P. FastTree: computing large minimum evolution trees with profiles instead of a distance matrix. Mol Biol Evol 2009;26:1641-50.

6 Bokulich N.A.; Kaehler B.D.; Rideout J.R.; et al. Optimizing taxonomic classification of marker-gene amplicon sequences with QIIME 2's q2-feature-classifier plugin. Microbiome 2018;6:90.

7 Koljalg U.; Nilsson R.H.; Abarenkov K.; et al. Towards a unified paradigm for sequence-based identification of fungi. Mol Ecol 2013;22:5271-7.
